# Editorial: Medical Students and Postgraduate Trainees as Medical Educators: peer learning is everywhere

**DOI:** 10.15694/mep.2017.000115

**Published:** 2017-07-03

**Authors:** Julie Browne, James Read

**Affiliations:** 1 Centre for Medical Education; 2 University of Plymouth

**Keywords:** peer learning

## Abstract

This article was migrated. The article was marked as recommended.

In this special issue we would like to encourage authors to contribute as many diverse understandings as possible of how learning occurs in situations where the teacher is, as Osler so memorably put it, a senior student anxious to help his or her juniors. So although we expect to see plenty of work on peer assisted learning programmes within clinical skills units or hospitals we would like to invite authors to be creative in their interpretation of what **Medical Students and Postgraduate Trainees as Medical Educators** means. In particular we’d like to advance the definition to include “teaching and learning with flat hierarchies” - that is to say, teaching and learning that takes place between people who are fairly close in age, experience or stage of learning, as well as teaching and learning that occurs in the ‘opposite direction’ - such as when seniors are able to learn from juniors. We will be celebrating the remarkable potential of peer teaching and learning with flat hierarchies and look forward to the publication of a variety of articles, including those that would not normally find an outlet elsewhere, such as replication studies, negative findings, pilots and protocols, case reports, narrative studies and reflections.

## Teaching and learning with flat hierarchies

*The successful teacher is no longer on a height, pumping knowledge at high pressure into passive receptacles..he is a senior student anxious to help his juniors.’ Sir William Osler (1849-1919)[1]*

Welcome to the latest themed edition of *MedEdPublish.* We have taken great interest in the development of the journal to date and it has been a real pleasure to see that so many of the submitted publications have been from junior educators involved in peer or near-peer teaching experiences. Medical students and postgraduate trainees have been active teachers within medical education for many years, but more recently there has been a greater interest in the tremendous potential of this approach to learning.

In this special issue, therefore, we would like to encourage authors to contribute as many diverse understandings as possible of how learning occurs in situations where the teacher is, as Osler so memorably put it, a senior student anxious to help his or her juniors. So although we expect to see plenty of work on peer assisted learning programmes within clinical skills units or hospitals we would like to make a particular call for authors to be creative in their interpretation of what **Medical Students and Postgraduate Trainees as Medical Educators** means. In particular we’d like to advance the definition to include “teaching and learning with flat hierarchies” - that is to say, teaching and learning that takes place between people who are fairly close in age, experience or stage of learning, as well as teaching and learning that occurs in the ‘opposite direction’ - such as when seniors are able to learn from juniors.

Flat hierarchies are not a new concept in areas outside of medical education. The term is widely used in the aviation industry, where the idea that more senior pilots are also your peers has been introduced to ensure that individuals feel able to speak up about issues which they feel might, for example, impact on the safety of the aeroplane or its occupants ( Schneider & Barbera 2014). Peer teaching draws upon this idea, where the concept of there being only a shallow ‘slope’ between teacher and learner aids in the exchange of learning and ideas between different people.

In April 2017 we were both involved in organising a national academic meeting at which a number of enthusiastic medical students and trainees presented a range of exciting and innovating perspectives on peer teaching, particularly the informal encounters that often occur outside the classroom in clinical settings, libraries or staff rooms. As we listened and learned we realised that peer teaching is everywhere - it is so much a part of medical education that it sometimes takes an effort even to see that it is going on alongside more formal teaching modalities.

‘Flat-hierarchy’ teaching and learning in medicine has a number of distinctive features that make it uniquely valuable in educational terms - while also making it a challenge to investigate. Here are our top six suggestions for the qualities that make peer learning and teaching so educationally rich - although there are almost certainly many more. We have conveniently organised them under the acronym ‘LUPINS’:

1. Learning focussed

When learners learn from and with other learners, the focus is likely to be far more on feedback and learning than assessment. Even where students are working together to study for an exam, they will be concentrating on exchanging ideas and understandings. Peer teaching is rarely part of the formally assessed curriculum, which reduces stress for the learners, but also makes them more relaxed about what and how they are learning and perhaps less inclined to conceal what they don’t know. Formative, on the spot feedback is a powerful tool for supporting deeper learning approaches, and flat hierarchy teaching is an excellent way to encourage the exchange of informal, targeted feedback ( Harrison et al 2016).

2. Unplanned, unrehearsed, unrecorded

This type of learning is often organised spontaneously, alongside the formal curriculum; such as when a student wants advice on writing a paper, a researcher needs to understand research ethics, or a group of junior doctors need to understand a clinical procedure. This often makes it relaxed and social, meaning that both learners and teachers may be more willing to question, innovate and collaborate to produce new understandings of the topic. There is less likely to be ‘performance anxiety’ on the part of the teacher in situations where the learning is a social, group activity rather than a formally assessed component of the curriculum.

3. Practical, patient focussed

Peer learning is usually based on clearly defined needs for information on a particular topic aimed at improving the learners’ care for patients: such as when a third year medical student asks a fifth year to help her with cannulation or a couple of specialty trainees ask a newly post CCT doctor to facilitate a session on a particular condition. This makes it powerful because it is driven by practical considerations that give it relevance and immediacy, and it is often at the specific request of the learner. In this way, learners develop an understanding of the value of autonomy and self-directedness as they take more responsibility for their learning (Williams and Deci 1998).

4. Immediate

Despite efforts to standardise peer teaching through programmatic approaches in medical schools and skills centres, most peer teaching is still a matter of taking advantage of immediate opportunities that arise in ‘live’ settings. It frequently happens in clinical environments with patients present, such as when a junior doctor is supervising a medical student to take a history or perform a procedure. It therefore requires teacher and learner to accept a degree of real-life uncertainty and imparts an immediacy to the learning that they do not experience in more formal teaching settings such as classrooms and simulation suites ( Bleakley and Bligh 2008).

5. Narrative based

Flat hierarchy learning usually involves narrative as the teacher and the learner seek shared experiences on which to base their learning. This is particularly true of situations where the learner identifies with the teacher as a role model. The conversation that ensues can be a valuable exchange of memorable insights into real-world clinical practice.

6. Small scale and social

Medical students and trainees are forever coming together and moving apart within teams and on placements; as they learn from and with each other, they form learning partnerships that coalesce and dissolve. As this activity progresses a number of other important things, such as teamwork, professional boundaries, social learning skills and time and resource management are being learned in addition to the subject matter ( Bleakley 2006).

Of course, the amazing educational potential of flat hierarchies in medical education is also what makes this area so hard to study and report. As John Spencer observes; “Clinical teaching has been much criticised for its variability, lack of intellectual challenge, and haphazard nature. In other words, clinical teaching is an educationally sound approach, all too frequently undermined by problems of implementation” ( Spencer 2003). These problems of implementation exemplify peer learning, which is such a characteristic of learning medicine and practising as a health care professional.

*MedEdPublish*, however, offers a unique opportunity to scholars interested in what happens when medical students and trainees learn with and from each other. The variability and ‘ordinariness’ of peer learning makes it hard to study and report, but in this next special issue we look forward to showing how scholars across the world are doing just that. We will be celebrating the remarkable potential of peer teaching and learning with flat hierarchies through articles that would not normally find an outlet elsewhere, such as replication studies, negative findings, pilots and protocols, case reports, narrative studies and reflections ( Hays 2016). By engaging with this exciting new publication model, scholars, students and practitioners in the field of peer teaching can present their work, appraise the evidence and discuss the issues they face with like-minded colleagues - in other words, they can actually engage in learning with and from their peers in a relevant, practical and constructive format that has been specially constructed to serve their needs. We look forward to hearing from you.

[1] Osler W. Aequanimitas, with other addresses to medical students, nurses and practitioners of medicine. Philadelphia: The Blakiston Company; 1910.

## Notes On Contributors

Julie Browne is a Senior Lecturer in Academic Practice at Cardiff University School of Medicine and course lead for the intercalated BSc in Medical Education. She is vice Chair of the Academy of Medical Educators Council and serves on a number of its standing committees including Education and Professional Standards. She is also a Senior Fellow of the Higher Education Academy. Her professional background is in academic publishing and she was formerly Managing Editor of *Medical Education* and *The Clinical Teacher.* In 2007 she co-ordinated the work of the Expert Advisory Panel on assessment for the Tooke Inquiry into Modernising Medical Careers. She is a GMC Education Associate, and is the author or co-author of a number of peer reviewed publications on medical education, including several chapters and a book on medical curriculum development. Her interests include educator development, medical humanities, publication ethics and curriculum theory. In 2015 she was awarded the President’s Silver Medal of the Academy of Medical Educators for outstanding and sustained contributions to medical education.

Dr Jamie Read is currently a PhD student with the CAMERA team at Plymouth University. Until recently he was an NIHR funded Academic Clinical Fellow with research interests in remediation in medical students and doctors and the role that near-peer education can play in both formal and informal training of medical students. His clinical background is as a trainee in Geriatric Medicine based in the South West of the UK, most recently at Derriford Hospital in Plymouth. Jamie has been involved in supporting early careers medical educators for some time and until recently he chaired the Early Careers Group of the Academy of Medical Educators - the professional organisation for educators of doctors, dentists and vets within the UK which has produced Professional Standards to support medical educators. He is now the Academy Registrar and Council member and retains a strong interest in encouraging more junior medical educators to develop their careers.

## Declarations

Julie Browne and James Read are guest Theme Editors for the AMEE MedEdPublish themed issue - Medical students and postgraduate trainees as medical educators.
