# Supplementary material for: Editorial: Medical Students and Postgraduate Trainees as Medical Educators: peer learning is everywhere
Source: MedEdPublish (2016). 2017 Jul 3;6:115. [Version 1] doi: 10.15694/mep.2017.000115 (PMC10885270; doi:10.15694/mep.2017.000115)
Supplement: Peer review history

# Reviewer report: Reviewer response for version 1

**DOI:** 10.21956/mep.19307.r28159

**Published:** 2019-04-10

**Authors:** Barbara Jennings

**Affiliations:** 1 Norwich Medical School

This review has been migrated. The reviewer awarded 4 stars out of 5 I very much agree with the title of this editorial. Peer-learning is everywhere in our medical schools, and I view it as a great driver of adult-learning, and of our agency as scientists, clinicians, and teachers.From peer review of scholarly articles, to peer observation of teaching events, and feedback or hypothesis-generation between PBL students; the value of a rigorous critical-friend seems clear. But as the editors say “the ordinariness of peer learning” can make it hard to study or to report in journals, despite a need to appraise it as an intervention (or as many interventions).The editors thoughtfully identify principles and contexts for peer-learning and I like their proposed definition of “teaching and learning with flat hierarchies”.This is an excellent topic to consider in an open access journal that encourages open conversation as part of its peer review process.

Reviewer Expertise:

NA

No decision status is available

# Reviewer report: Reviewer response for version 1

**DOI:** 10.21956/mep.19307.r28158

**Published:** 2017-07-06

**Authors:** Trevor Gibbs

**Affiliations:** 1 AMEE

This review has been migrated. The reviewer awarded 5 stars out of 5 For far too long, teaching has been " what it says on the tin"- a one-way process from expert to learner with as minimal an interaction as possible and based upon filling the head of the student with the "knowledge" that the teacher is prepared to share. As years have progressed, we have seen the fantastic development of a more student-centred approach to the two way process of learning; of making the learning more active, integrated and relevant and socially accountable. However, it frequently remains a hierarchical structure, a top-down approach with curricula designed and formed by more senior educators. I am looking forward to reading the papers in this theme which looks at this "flat hierarchy", of using peer, near-peer and other forms of students using their skills to promote learning amongst others. This remains an un=tapped resource, which is going to be vital as we become more resource-constrained, for whatever reason. Like the authors I too believe that there is a host of other teaching and learning activities out there and would encourage all potential authors to share their innovations.

Reviewer Expertise:

NA

No decision status is available
